# The SUMO E3 Ligase MdSIZ1 Sumoylates a Cell Number Regulator MdCNR8 to Control Organ Size

**DOI:** 10.3389/fpls.2022.836935

**Published:** 2022-04-15

**Authors:** Gui-Luan Wang, Chun-Ling Zhang, He-Qiang Huo, Xiao-Shuai Sun, Ya-Li Zhang, Yu-Jin Hao, Chun-Xiang You

**Affiliations:** ^1^State Key Laboratory of Crop Biology, College of Horticulture Science and Engineering, Shandong Agricultural University, Taian, Shandong, China; ^2^Mid-Florida Research and Education Center, University of Florida, Institute of Food and Agricultural Sciences, Apopka, FL, United States; ^3^College of Science, Tibet University, Lhasa, China

**Keywords:** cell number, MdCNR8, MdSIZ1, organ size, root growth, SUMOylation

## Abstract

Plant growth and organ size putatively associated with crop yield are regulated by a complex network of genes including ones for controlling cell proliferation. The gene *fw2.2* was first identified in tomatoes and reported to govern fruit size variation through controlling cell division. In this study, we isolated a putative ortholog of the tomato *fw2.2* gene from apple, *Cell Number Regulator 8* (*MdCNR8*). Our functional analysis showed that *MdCNR8* may control fruit size and root growth. MdCNR8 was mediated by the SUMO E3 ligase MdSIZ1, and SUMOylation of MdCNR8 at residue-Lys39 promoted the translocation of MdCNR8 from plasma membrane to the nucleus. The effect of *MdCNR8* in inhibiting root elongation could be completely counteracted by the coexpression of *MdSIZ1*. Moreover, the lower cell proliferation of apple calli due to silencing MdSIZ1 could be rescued by silencing MdCNR8. Collectively, our results showed that the MdSIZ1-mediated SUMOylation is required for the fulfillment of MdCNR8 in regulating cell proliferation to control plant organ size. This regulatory interaction between *MdSIZ1* and *MdCNR8* will facilitate understanding the mechanism underlying the regulation of organ size.

## Introduction

Plant organ size can significantly vary in natural populations within species. The variation in plant organ size within a species could be contributed by the evolving genetic factors that enable plants to adapt the changing environment and artificial selection of human being for seeking desirable agronomic traits (Mizukami, 2001; Weiss et al., 2005). Despite that increase in cell number rather than cell size has been previously proposed to be associated with large plant organ size (Guo et al., 2010), the recent studies have uncovered key regulatory genes that affect plant organ size by altering both cell number and cell size (Powell and Lenhard, 2012).

Several genetic factors have been identified for controlling plant organ size. In Arabidopsis, *AUXIN REGULATED GENE INVOLVED IN ORGAN SIZE* (*ARGOS*) is a gene encoding ER-localized protein of unknown function. Overexpression of *ARGOS* promoted cell proliferation and resulted in larger aerial organs (Hu et al., 2003). *ARGOS* promotes cell proliferation through stimulating expression of an AP2/ERF transcription factor *AINTEGUMENTA* (*ANT*), which in turn maintains the expression of the D-type cyclin *CYCD3;1* for cell division (Hu et al., 2003). In addition, the transcription factors of the TEOSINTE BRANCHED1, CYCLOIDEA, PCFs (TCP) and GROWTH-REGULATING FACTOR (GRF) involve in regulating cell proliferation to control organ size (Powell and Lenhard, 2012). The TCP genes can be targeted and downregulated by a microRNA, miR319a. Additionally, the TCP4 promotes the expression of another microRNA, miR396, which targets seven of the nine members of the GRF gene family. Over-accumulation of T due to the loss of function in miR319a or dysfunction of GRFs resulted in smaller organ size (Schommer et al., 2014). In a parallel pathway, the putative ubiquitin-binding protein DA1 and the E3 ubiquitin-ligase Big Brother limit organ size by suppressing cell proliferation (Li et al., 2008). Yet, the molecular mechanisms responsible for cell proliferation and cell expansion are still poorly understood.

Another key regulator for controlling organ size is the FW2.2 like protein. The *SlFW2.2* gene was the first identified for controlling fruit size in tomato (*Solanum lycopersicum*) (Frary et al., 2000). *SlFW2.2* encodes a repressor of cell division during tomato fruit development, and increased expression is associated with the reduction in cell division and fruit size (Frary et al., 2000; Cong et al., 2002). The recent studies also showed that *FW2.2* is conserved in regulating organ size across different plant species, and homologous *FW2.2-like* genes have been identified in several species, which includes *Pafw2.2-like* in avocado (*Persea americana*), *GmFWL1* in soybean (*Glycine max*), *Osfwl3* in rice (*Oryza sativa*), *Cell Number Regulator 1 (CNRs*) in maize (*Zea mays*), cherry (*Prunus avium*), and *Physalis floridana* (Dahan et al., 2010; Guo et al., 2010; Libault et al., 2010; De Franceschi et al., 2013; Xu et al., 2013; Li and He, 2015). All these *FW2.2-like* genes play a conservative role in repressing cell division to exert their function in controlling plant organ size.

To date, less is known about the molecular and biochemical role of these FW2.2 proteins in cell division (Guo and Simmons, 2011; Van Der Knaap et al., 2014). The previous studies suggested that FW2.2-like proteins may facilitate transporting of cadmium and calcium ions (Song et al., 2004; Nakagawa et al., 2007), but it is very perplexing how its function in ion changes is coupled with regulating cell division. A yeast two-hybrid (Y2H) assay revealed that SlFW2.2 could interact with the regulatory subunit of casein kinase II (CKII) (Cong and Tanksley, 2006) that has a role in the control of cell division (Pepperkok et al., 1994; Espunya et al., 1999; Moreno-Romero et al., 2008). In addition, a recent report showed that the FW2.2-like protein PfCNR1 in *Physalis floridana* was able to interact with a putative MADS-domain transcription factor PfAG2 and colocalized in the nuclei (Li and He, 2015), which also raised questions of how PfCNR1 was transported to the nucleus and whether its nuclear transport is assisted by any other proteins.

SUMOylation is a post-translational modification in which SUMO (small ubiquitin-like modifier) peptides are covalently attached to specific lysine residues in target proteins (Sampson et al., 2001; Schmidt and Muller, 2003; Seeler and Dejean, 2003). The SUMOylation process begins with a biochemical step of SUMO activation by a heterodimeric E1 complex consisting of a SUMO-activating enzyme such as SAE1 or SAE2 (SUMO E1); the activated SUMOs are subsequently transferred to a SUMO-conjugating enzyme SCE1 (SUMO E2) and finally conjugated to the substrate proteins with the help of the E3 type ligases SIZ1 and HPY2/MMS21 (SUMO E3) (Desterro et al., 1999; Bernier-Villamor et al., 2002; Novatchkova et al., 2004; Ishida et al., 2012; Novatchkova et al., 2012; Mazur et al., 2017). SUMOylation can cause alterations in stability, interaction, cellular localization, and activity of proteins and thereby regulates biological and biochemical processes such as nuclear transport (Stade et al., 2002; Sehat et al., 2010; Lutz et al., 2012; Berkholz et al., 2014) and transcriptional regulation (Ross et al., 2002; Sapetschnig et al., 2002). The previous results on CNR-like proteins from different plant species may interact with nuclear-localized proteins to exercise their functions (Cong and Tanksley, 2006; Li and He, 2015), and it is plausible to speculate that cellular translocation of MdCNR8 exists and that this event may be mediated by SUMOylation.

In this study, we reported that MdCNR8 can be sumoylated by MdSCE1 and MdSIZ1 at the lysine 39 for cellular translocation to control root growth and fruit size. Our results presented here may provide novel insights into understanding the molecular mechanism by which FW2.2-like gene controls organ size.

## Materials and Methods

### Plant Materials

Plant materials used in this study that include wide-type Arabidopsis seedlings (Col-0), wide-type AC (*Ailsa Craig*) tomato seedlings (*Solanum lycopersicum*), the wide-type apple “Orin” calli (Xie et al., 2012), and wild-type (WT) apple seedlings “Gala” were maintained in our laboratory at Shandong Agricultural University.

### Vector Construction and Genetic Transformation

The coding regions of *MdSIZ1* and *MdCNR8* were amplified from cDNA with primers (Supplementary Table 1) designed based on the gene sequences (*XM_008387065* for *MdSIZ1* and *XM_008371558* for *MdCNR8*). cDNA was synthesized by a PrimeScript First-Strand cDNA Synthesis Kit (Takara, Dalian, China). The amplified *MdSIZ1* and *MdCNR8* fragments were inserted into the pRI101AN vector for binary expression constructs CaMV 35S:*MdSIZ1-GFP* and CaMV 35S:*MdCNR8-GFP*, which were transformed into *Agrobacterium tumefaciens* strains LBA4404 and GV3101 or *Agrobacterium rhizogenes* K599 for plant genetic transformation.

Arabidopsis plants were transformed using *Agrobacterium* (GV3101)-mediated floral dip method to obtain *35S:MdSIZ1-GFP*, *35S:MdCNR8-GFP* overexpression lines (Clough and Bent, 1998). The double overexpression lines of *MdSIZ1* and *MdCNR8* were obtained by crossing both single expression lines. T3 homozygous Arabidopsis plants were used for all the analyses in this study. Arabidopsis seeds were germinated and cultured vertically on 1/2 MS medium under 16-h light/8-h dark at 23°C for 10 days for root growth observation and cell number measurement.

Genetic transformation of tomato was performed using *Agrobacterium* (LBA4404)-mediated method as described (Sun et al., 2006). For the measurement of fruit characteristics, the fruits with the same developmental stages were harvested to determine their size (height and width) and weight.

Prior to genetic transformation, the WT apple calli were pre-cultured on MS medium containing 3 mg⋅L^–1^ 2, 4-D and 0.4 mg⋅L^–1^ 6-BA at 25°C in the dark for 20 days. The apple calli were subsequently infected by *Agrobacterium* (LBA4404) with the method as described previously (Sun et al., 2018). These transgenic calli were used for protein extraction and gene expression analysis. The WT apple calli were used for SUMOylation assay and plasma membrane protein isolation.

Apple hair root transformation was performed using *Agrobacterium* (K599)-mediated method as described (Ma et al., 2019b).

The “*Nicotiana benthamiana*” tobacco plants were grown under 16-h light/8-h dark conditions at 25°C and 65% humidity for 30 days for *Agrobacterium* (LBA4404) infection for BiFC assays and fluorescence observation.

### Gene Expression Analysis

Total RNAs were isolated from apple seedlings, apple calli, Arabidopsis seedlings, and tomato seedlings using RNA plant plus (TIANGEN, Beijing, China). First-strand cDNA was synthesized using PrimeScript RT Reagent Kit (Takara, Dalian, China). Primers (Supplementary Table 1) for qRT-PCR were designed using DNAClub software, and the transcript level of different genes detected with the BIO-RAD IQ5 system (Bio-Rad, Hercules, CA). Three biological replications and three technical replications were applied to each sample. The relative transcript level was normalized with a reference gene *MdACTIN*.

### Screening of T-DNA Insertion Mutants

*atcnr8* mutant seeds (SALK_042402) were purchased from the T-DNA insertion mutant library. The primers were designed by the website^1^, and homozygotes were screened out using the three-primer method.

### Yeast Hybrid Assays

Yeast (*Saccharomyces cerevisiae*) two-hybrid assays were carried out as described (An et al., 2018). The *MdCNR8, MdCNR8^1–92^*, *MdCNR^93–246^*, and *AtCNR8* coding sequences were cloned into pGBT9 vector. The full-length *MdSIZ1* and *AtSCE1* genes were cloned into the pGAD424 vector. The recombinant vectors were also transformed into Gold yeast strain, and then, the yeast strains were cultured on the SD (-T/-L/) medium, SD (-T/-L/-H/-A) medium, and SD (-T/-L/-H/-A) medium added to X-gal.

Yeast three-hybrid assays were performed following the previously described protocol (Licitra and Liu, 1996; Lv et al., 2019). The *MdCNR8* coding sequence was cloned into pGADT7 vector. The *MdSIZ1-N* and *MdSCE1* genes were cloned into pBridge vector (MdSCE1 as bridge protein). The recombinant vectors were also transformed into Gold yeast strain, and then, the yeast strains were cultured on the SD (-Trp/-Leu) medium and SD (-Trp/-Leu/-His/-Met) medium. The *pBridge-MdSIZ1-N* and *pGADT7* was used as the negative control.

### Pull-Down Assays

The coding region of *MdCNR8* was cloned into *PET32a* vector, and the coding region of *MdSCE1* was cloned into *PGEX 4X-1* vector. The recombinant vectors of *MdCNR8-PET32a* and *MdSCE1-PGEX 4X-1* and empty vector of *PGEX 4X-1* were transformed into *Escherichia coli* BL21 (TransGen, Beijing, China). To produce glutathione-S-transferase (GST) and His-fusion protein, we treated *Escherichia coli* BL21 with 0.1-mm isopropyl β-D-1-thiogalactopyranoside for 6 h at 37°C. Pull-down assays were carried out as previously described (Ma et al., 2019a). Finally, we detected the pellet fraction *via* immunoblotting with anti-His and anti-GST antibody (Abmart, Shanghai, China).

### Bimolecular Fluorescence Complementation Assays

MdSCE1-YFP^N^ and MdCNR8-YFP^C^ recombinant vectors were constructed as described above and transformed into the *Agrobacterium* LBA4404 for the infiltration of tobacco leaves. The agrobacteria mixture of MdSCE1-YFP^N^ and MdCNR8-YFP^C^, MdSCE1-YFP^N^ and YFP^C^, and MdCNR8-YFP^C^ and YFP^N^ was injected tobacco epidermal cells for fluorescent imaging with a Zeiss confocal laser scanning microscope.

### SUMOylation Assays

SUMOylation assay *in vitro* was performed in a 50-μl reaction system containing 50 mm Tris-HCl, 100 mm NaCl, 15% glycerol, 5 mm adenosine triphosphate (ATP), 10 mm MgCl_2_, pH 7.8, 5 μg SUMO1 protein (Abcam, Cambridge, United Kingdom), E1 (0.5 μg of human SAE1 full and 0.5 μg of SAE2/UBA2 peptide), E2 (2 μg of MdSCE1/2 μg of human UBE2/UBC9 peptide), 8 μg of MdSIZ1 protein, and purified MdCNR8-His protein. ATP was not added in this system as a negative control. After incubation for 6 h at 37°C, sumoylated His-MdCNR8 was detected using western blot by anti-His antibody and anti-SUMO1 antibody.

SUMOylation assay *in vivo* was performed using transgenic MdCNR8-FLAG, MdCNR8^K39R^-FLAG, MdCNR8-FLAG/MdSIZ1-GFP, and MdCNR8-FLAG/antiMdSIZ1-GFP apple calli. Proteins were extracted from these calli, and MdCNR8-FLAG and MdCNR8^K39R^-FLAG proteins were immunoprecipitated using a Pierce Classic IP Kit (Thermo, Shanghai, China) with an anti-FLAG antibody. Sumoylated MdCNR8-FLAG was detected by western blot with anti-FLAG antibody and anti-SUMO1 antibody. MdSIZ1-GFP was detected by western blot with anti-SIZ1 antibody (customized by Abmart). ACTIN was used as a sampling quantity control.

### Confocal Microscopy Analysis

The green fluorescent protein (GFP), red fluorescent protein (RFP), and yellow fluorescent protein (YFP) were imaged under a Zeiss confocal laser scanning microscope. The process was carried out as previously described (Li et al., 2017a; Lv et al., 2019). Approximately 10 seedlings/images per experiment were examined, and at least three independent experiments were performed for the statistical significance analysis.

### Protein Extractions

BestBio kits (BB-3169 and BB3152) were used to extract the proteins from cell nuclei, plasma membrane, and cytoplasm. Protein concentration was measured by the BCA method (Sangon C503061). Protein extracts were analyzed by immunoblotting with anti-Flag (Abmart, China), anti-APX3 (PhytoAB, United States), anti-Actin (Abcam, United Kingdom), and anti-Histone H3 (Abcam, United Kingdom) antibodies.

## Results

### MdCNR8 Regulates Organ Size by Negatively Regulating the Cell Proliferation

**FIGURE 1:**
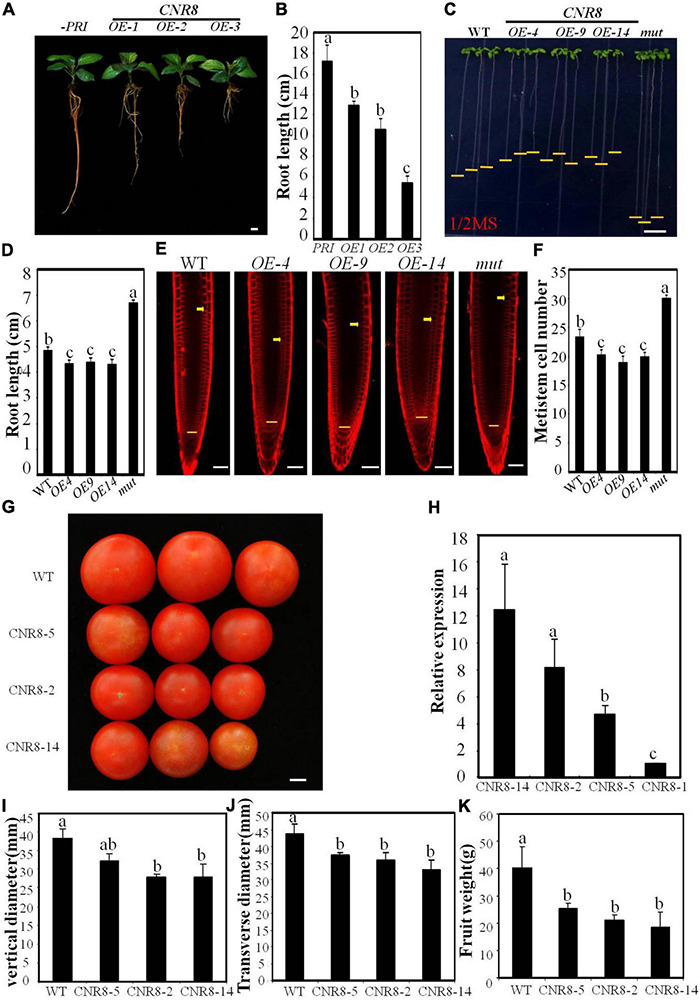
Phenotypes of *MdCNR8* transgenic lines. **(A)** The 40-day-old seedlings of *MdCNR8* overexpression lines (OE-1, OE-2, and OE-3) and the control line infected with empty vector. Bars = 1 cm. **(B)** Primary root length of 40-day-old apple seedlings of empty control, OE1, OE2, and OE3. Mean values were shown with their SD (*n* = 11 to 20). **(C)** Root phenotypes of the WT, *MdCNR8* overexpression lines (OE-4, OE-9, and OE-14) and *atcnr8* mutant (mut) grown for 7 days on 1/2 MS medium. Bars = 1 cm. **(D)** Primary root length of the 7-day-old Arabidopsis seedlings of WT, OE-4, OE-9, OE-14, and *atcnr8* mutant. Mean values were shown with their SD (*n* = 11 to 20). **(E)** Confocal microscopy images of the FM4-64-stained Arabidopsis seedlings of WT, *MdCNR8* overexpression lines (OE-4, OE-9, OE-14) and *atcnr8* mutant (mut). Yellow arrows and horizontal lines indicate the approximate position of root meristem zone. Bars = 50 μm. **(F)** Meristem cell number of Arabidopsis seedlings of WT, OE-4, OE-9, OE-14, and *atcnr8* mutant (mut). Cell number of 7-day-old roots was counted and shown as averages ± SD (*n* = 5 to 10). **(G)** Mature tomato fruit phenotypes of the WT and *MdCNR8* overexpression lines (OE-2, OE-5, OE-14). Bars = 1 cm. **(H)** qRT-PCR of *MdCNR8* in tomato plants. Data are the means ± SD of three biological repeats. *SlACTIN* was used as an internal control. **(I–K)** Quantification of vertical diameter, transverse diameter and fruit weight of the WT, OE-2, OE-5, and OE-14 in **(G)**. Different letters on each bars denote significant differences (*p* < 0.01, ANOVA, Tukey’s correction).

To understand the genetic control of fruit size in apple, we have isolated a homolog of *SlFW2.2, MdCNR8* from apple. The *MdCNR8* has two introns and three exons, which shows 67.5% protein similarity to tomato SlFW2.2-like (SlCNR8). To examine tissue-specific expression of MdCNR8, its transcript levels were analyzed in the root, stem, young and mature leaf, axillary bud, floral bud, flower, petal, stamen, ovary, sepal, fruit, fruit skin, seed, and pulp using a quantitative reverse transcription polymerase chain reaction (qRT-PCR) assay. As shown in Supplementary Figure 1, MdCNR8 was predominantly expressed in fruit skin of developing fruit and young leaf, and the lowest level of MdCNR8 was observed in axillary bud, floral bud flower, unfertilized ovary, and sepal. To characterize its function, we next overexpressed *MdCNR8-GFP* in apple roots through *Agrobacterium rhizogenes* infection. The strong GFP fluorescence and GFP protein abundance in roots of three overexpression lines indicated that the fused *MdCNR8-GFP* gene was in-frame translated and functional in hairy roots (Supplementary Figures 2A,B). Interestingly, the root elongation was significantly decreased in *MdCNR8* overexpression lines compared to the control line infected with empty vector (Figures 1A,B). To further examine the role of *MdCNR8* in plant development, we generated transgenic Arabidopsis-overexpressing *MdCNR8-GFP*. Based on the *MdCNR8* transcript level determined by qRT-PCR (Supplementary Figure 2C), three *MdCNR8-GFP* Arabidopsis overexpression lines such as OE-4, OE-9, and OE-14 were chosen for further analysis. In addition, a homozygous Arabidopsis T-DNA mutant *cnr8* (*SALK_042402*) with undetectable endogenous *CNR8* transcript was also included for comparisons (Supplementary Figure 2D). It was found that *MdCNR8* overexpression can partially rescue the root length phenotype of *atcnr8* by statistical analysis of WT, *atcnr8* and *MdCNR8*/*atcnr8* root length, which indicates that *MdCNR8* and *AtCNR8* have similar functions (Supplementary Figures 2E,F). Similar to *MdCNR8* overexpression in apple roots, expression of *MdCNR8* in Arabidopsis also caused a significant reduction in root growth, whereas root elongation was enhanced in the Arabidopsis *cnr8* mutant (Figures 1C,D). These results suggested that *MdCNR8* negatively regulates root growth. The previous results from other plant species may imply that *MdCNR8* putatively plays a role in cell division, and thus, we examined the number of meristematic cells stained with FM4-64. The meristematic cell number of *MdCNR8*-overexpressed seedlings was less than WT, whereas the meristematic cell number of mutants was significantly more than WT (Figures 1E,F). These results indicated that *MdCNR8* suppresses cell proliferation in the root meristematic zone.

To determine whether *MdCNR8* functions in controlling fruit size, we generated transgenic tomato plants overexpressing *MdCNR8*-*GFP* in tomato [*S.lycopersicum* cv. *Ailsa Craig* (AC)]. Based on the *MdCNR8* transcript level determined by qRT-PCR (Figure 1H), three *MdCNR8-GFP* tomato overexpression lines such as OE-2, OE-5, and OE-14 were chosen for further analysis. The fruit size of *MdCNR8*-overexpressing tomato was significantly smaller than WT (Figure 1G), which was further supported by the results of vertical diameter, transverse diameter, and fruit weight (Figures 1I–K). Collectively, all these results indicated that *MdCNR8* regulates organ size through negatively mediating cell proliferation.

### MdCNR8 Interacts With MdSCE1

**FIGURE 2:**
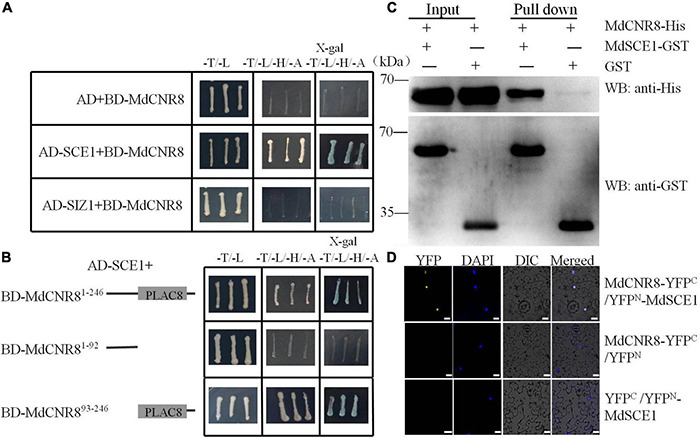
MdCNR8 interacts with MdSCE1. **(A)** Interaction of MdCNR8 with MdSCE1 as shown by Y2H assays. The full-length MdCNR8 was cloned into vector pGBT9 (BD-MdCNR8). The MdSCE1 or MdSIZ1 was cloned into pGAD424 (AD-MdSCE1/AD-MdSIZ1). Empty vector AD + BD-MdCNR8 was used as a negative control. SD/-T/-L indicates synthetically defined medium lacking Leu and Trp; SD/-T/-L/-H/-A indicates synthetically defined medium lacking Leu, Trp, His, and Ade. x-gal, 5-Bromo-4-chloro-3-indolyl β-D-galactopyranoside. **(B)** Y2H segmentation assays for the interaction between the C-terminus containing PLAC8 conservative motif of MdCNR8 and MdSCE1. MdCNR8^1–92^ and MdCNR8^93–246^ vectors were fused to pGBT9 vector. **(C)** Pull-down assays for the interaction of MdSCE1 with MdCNR8. *Escherichia coli*-expressed GST or MdSCE1-GST proteins were incubated with a cobalt chelate affinity resin containing the immobilized histidine-tagged MdCNR8 protein. The proteins were detected *via* immunoblotting with anti-His and anti-GST antibody. **(D)** Biomolecular fluorescence complementation assays for the interaction between MdSCE1 and MdCNR8 proteins in nuclei of epidermal cells of tobacco. DAPI (4′,6-diamidino-2-phenylindole) was used to stain the nuclei. YFP, yellow fluorescent protein; differential interference contrast (DIC), bright field. Bars = 20 μm.

Based on the computational prediction, *MdCNR8* contains one transmembrane motif, which suggests that it might be a membrane-localized protein as FW2.2-like proteins in other plant species (Guo et al., 2010; De Franceschi et al., 2013; Qiao et al., 2017). Although the PfCNR1 protein in *P. floridana* has been proposed to interact with a PfAG2 protein to exert its repressive role in cell division (Li and He, 2015), the failure in detection of interaction between PfCNR1 and PfAG2 *in vivo* made it difficult to explain how PfCNR1 mediates cell division through affecting the function of PfAG2. In addition, it is still not clear whether a post-translational modification occurs to MdCNR8 for exerting its function. Therefore, we performed Y2H assay for screening whether any other MdCNR8-interacting proteins exist in apple. We used MdCNR8 as a bait to screen the apple cDNA library. As a result, several positive colonies were obtained, and one of them contained a part of cDNA fragment of MdSCE1, a SUMO-conjugating enzyme. The independent Y2H assays have confirmed the interaction of MdSCE1 and MdCNR8 (Figure 2A). SCE1 and SIZ1 (a SUMO E3 ligase) are the two essential components for protein SUMOylation and interacts with each other. We next examined whether MdSIZ1 also directly interacts with MdCNR8. The result from Figure 2A and Supplementary Figure 3 showed that there is no direct interaction between MdSIZ1 and MdCNR8.

MdCNR8 contains a PLAC8 domain (93-216aa) at its C-terminus. To examine whether the PLAC8 domain is required for the MdSCE1-MdCNR8 interaction, an N-terminal section without the PLAC8 domain and a C-terminal region containing the PLAC8 domain were used for examining their interactions with MdSCE1. As shown in Figure 2B, BD-MdSCE1 only interacted with the C-terminal not the N-terminal region (Figure 2B), which suggests that the PLAC8 domain is essential for MdSCE1-MdCNR8 interaction. The robust interaction between MdCNR8 and MdSCE1 was further confirmed by an *in vitro* pull-down assay. The His-tagged MdCNR8 could be cocaptured with a GST-tagged MdSCE1 as a protein complex when a GST bait protein was applied for a pull-down (Figure 2C), which further supports that MdCNR8 was able to interact with MdSCE1 *in vitro*. Furthermore, we performed a BiFC assay to examine whether this interaction also occurs *in vivo*. The fluorescent signal derived from the successful re-construction of YFP protein indicated that there was a strong interaction between MdCNR8-YFP^C^ and YFP^N^-MdSCE1 (Figure 2D).

### MdCNR8 Forms a Complex With MdSIZ1 and MdSCE1

**FIGURE 3:**
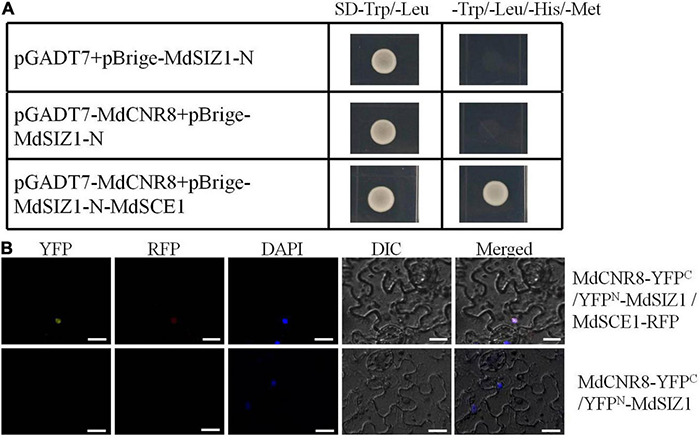
MdCNR8 forms a complex with MdSIZ1 and MdSCE1. **(A)** Yeast three-hybrid assay for the interaction of MdSIZ1, MdCNR8, and MdSCE1. The *MdCNR8* was cloned into pGADT7 vector (pGADT7-MdCNR8). The *MdSIZ1-N* and *MdSCE1* were cloned into pBridge vector (pBrige-MdSIZ1-N and pBrige-MdSIZ1-N-MdSCE1). pGADT7 + pBrige-MdSIZ1-N were used as a negative control. SD, synthetically defined medium. **(B)** Bimolecular fluorescence complementation assays for colocalization of MdSIZ1 and MdCNR8 in the nucleus of tobacco leaves cell with the participation of MdSCE1. DAPI was used to stain the nuclei. YFP, yellow fluorescent protein; RFP, Red fluorescent protein; DIC, bright field. Bars = 20 μm.

MdCNR8 was able to directly interact with MdSCE1 but not MdSIZ1 motivated us to explore whether MdSIZ1 as a SUMO E3 ligase was involved in SUMOylation of MdCNR8 through a protein complex with MdSCE1. A yeast three-hybrid assay was then performed and revealed that only the transgenic yeast with pGADT7-MdCNR8/pBrige-MdSIZ1-N-MdSCE1 recombinant vector could normally grow on the SD-Trp/-Leu/-His/-Met medium, which indicates that MdSIZ1 and MdCNR8 could interact with each other when MdSCE1 was used as a bridge protein (Figure 3A); thus, we concluded that MdCNR8 may form a protein complex with MdSIZ1 and MdSCE1 *in vitro*.

To examine whether this protein complex consisting of three proteins may exist *in vivo*, we performed another BiFC assay to verify the *in vivo* interaction of MdSIZ1, MdCNR8, and MdSCE1 in tobacco leaves. As the results shown in Figure 3B, a strong YFP signal was observed in the epidermal cells of tobacco leaves transformed with MdCNR8-YFP^C^, YFP^N^-MdSIZ1, and MdSCE1-RFP, but not leaves transformed with MdCNR8-YFP^C^ and YFP^N^-MdSIZ1, which indicates that the MdSCE1 is required for a successful protein complex formation of these three proteins (Figure 3B).

### MdCNR8 Can Be Sumoylated *in vivo* and *in vitro* and K39 Is Essential for SUMOylation of MdCNR8

**FIGURE 4:**
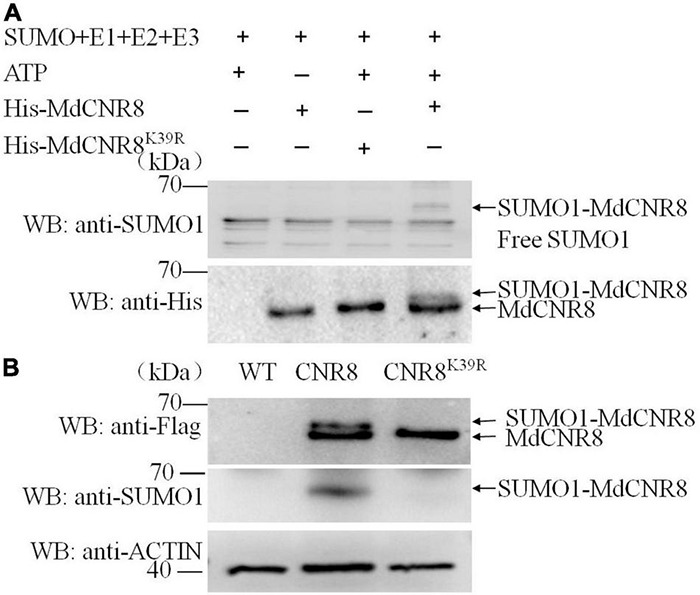
MdCNR8 can be sumoylated *in vivo* and *in vitro* and Lysine 39 is an essential SUMOylation site. **(A)** Detection of SUMO1-MdCNR8 conjugates *in vitro*. SUMO1-MdCNR8 conjugates were detected in the presence (+) or absence (–) of ATP, MdCNR8 or MdCNR8^K39R^ with anti-His and an anti-SUMO1 antibodies. **(B)** Detection of SUMO1-MdCNR8 conjugates *in vivo*. The proteins from WT, MdCNR8-FLAG, and MdCNR8^K39R^-FLAG calli were immunoprecipitated with anti-FLAG antibody. The proteins were detected with anti-FLAG antibody and anti-SUMO1 antibody. ACTIN was used as a control.

Considering the function of MdSCE1 and MdSIZ1 in the process of protein SUMOylation (Zhou et al., 2017), and they can interact with MdCNR8, we hypothesize that the MdCNR8 protein could be sumoylated. An *in vitro* SUMOylation assay was subsequently carried out to validate our hypothesis. Recombinant His-MdCNR8 was incubated with SUMO-activating enzyme E1, SUMO-conjugating enzyme E2, SUMO1, and SIZ1, followed by immunoblot analysis using anti-SUMO1 antibody and anti-His antibody. SUMO1-MdCNR8 conjugates were not detectable with anti-SUMO1 antibody in the absence of ATP or MdCNR8, but were detectable when all components were present. The SUMOylation of MdCNR8 resulted in high molecular weight and a slow mobility shift compared to the non-sumoylated MdCNR8 (Figure 4A).

Subsequently, the putative SUMOylation site consensus sequence φ-K-X-E/D (φ represents a hydrophobic amino acid, K represents a lysine that is conjugated to SUMO, X represents any amino acid, and E/D represents an acidic residue) (Sampson et al., 2001) was identified in MdCNR8 using SUMOplot^2^ analysis (Lin et al., 2016). The lysine 39 (K39) of MdCNR8 was conserved among different plant species and predicted to be SUMOylation site. To validate this prediction, we performed *in vitro* SUMOylation assay of MdCNR8^K39R^. SUMO1-MdCNR8 conjugates could not be detected with anti-SUMO1 and anti-His antibody in the presence of MdCNR8^K39R^, which is different from the result with MdCNR8 (Figure 4A). These results indicated that the SUMOylation of MdCNR8 is dependent on the lysine 39. In addition, we also performed *in vivo* SUMOylation assay. Total proteins were extracted from apple calli transformed with either *MdCNR8-FLAG* or a mutated *MdCNR8* with a conversion from lysine 39 to arginine *(MdCNR8^K39R^-FLAG)*. Both MdCNR8-FLAG and MdCNR8^K39R^-FLAG proteins were detected from the total apple calli protein using an anti-FLAG antibody by immunoblotting, but the size of MdCNR8-FLAG protein was obviously larger than MdCNR8^K39R^-FLAG protein due to SUMOs attached to MdCNR8-FLAG but not the mutated MdCNR8^K39R^-FLAG protein (Figure 4B); the attached SUMOs of MdCNR8-FLAG were also confirmed by the immunoblotting with an anti-SUMO antibody (Figure 4B), which suggests that K39 is essential for SUMOylation of MdCNR8 *in vivo*. In addition, we found that SUMO1-MdCNR8 can also be detected with anti-SUMO1 antibody and anti-His antibody in transgenic MdCNR-FLAG/MdSIZ1-GFP calli but not in MdCNR-FLAG/antiMdSIZ1-GFP calli, which indicates that MdSIZ1 is necessary for the SUMOylation of MdCNR8 in plants (Supplementary Figure 4).

### MdSIZ1-Mediated SUMOylation Increases Nuclear Localization of MdCNR8

**FIGURE 5:**
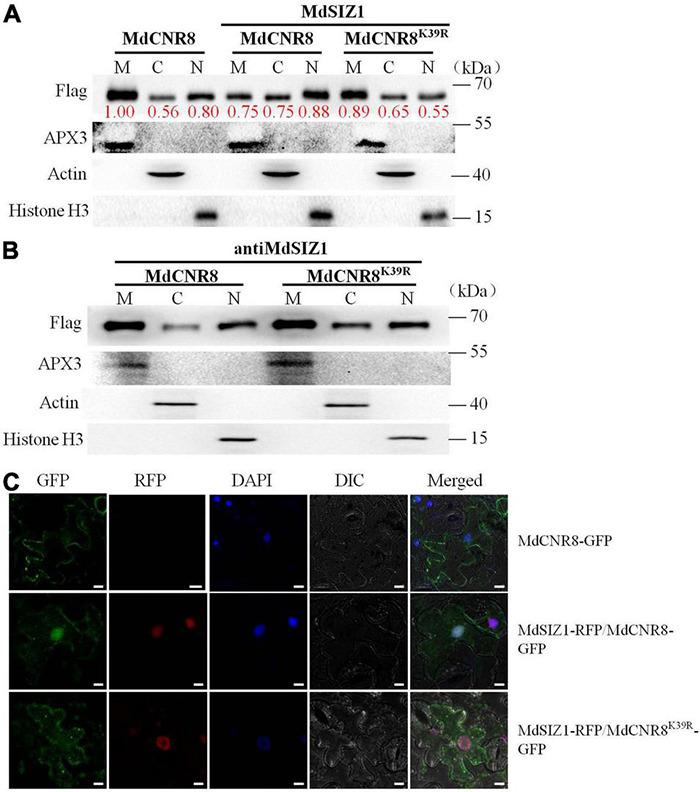
MdSIZ1 increases nuclear localization of MdCNR8. **(A,B)** The abundance of MdCNR8 or MdCNR8^K39R^ separated from cell membrane, cytoplasm, and nuclei in the presence **(A)** or absence **(B)** of MdSIZ1-GFP. M, cell membrane; C, cytoplasm; N, cell nuclei. The red numbers indicate the quantification of the protein band brightness. **(C)** Fluorescent detection of MdCNR8 subcellular localization in tobacco leaves. MdCNR8-GFP only or MdSIZ1-RFP combined with either MdCNR8-GFP or MdCNR8^K39R^-GFP constructs were coexpressed transiently in tobacco leaves and visualized by fluorescence microscopy. DAPI was used to stain the nuclei. GFP, green fluorescent protein; RFP, red fluorescent protein; DIC, bright field. Bars = 20 μm.

Interestingly, the BiFC assay revealed that MdSIZ1 and MdCNR8 were colocalized within the nucleus with the participation of MdSCE1 (Figure 3B), which raised an intriguing question of whether an alteration in cellular localization of MdCNR8 occurs when it is sumoylated. The previous studies have also demonstrated that SUMOylation may cause localization transfer of a target protein to nuclei (Sehat et al., 2010; Lutz et al., 2012; Liu et al., 2018). To address whether SUMOylation promoted the nuclear recruitment of MdCNR8, we first analyzed the abundance of MdCNR8 or MdCNR8^K39R^ in the context of the overabundance of MdSIZ1 using immunofluorescence staining and cell fractionation followed by a western blotting (Figure 5). We separated specific proteins from cell membrane, cytoplasm, and nucleus of apple calli transformed with *MdCNR8-FLAG* only or MdSIZ1 combined with either *MdCNR8-FLAG* or *MdCNR8^K39R^-FLAG*, respectively. The blotting results with antibodies against cytoplasm-specific protein ACTIN, nucleus-specific protein Histone H3, or membrane-specific protein APX3 confirmed that there was no cross-contamination between each specific protein isolate. In the apple calli transformed with *MdCNR8-FLAG* only, MdCNR8 is predominantly localized in the cell membrane compared with the proteins fractioned from cytoplasm and nuclei (Figure 5A). However, overexpression of MdSIZ1 increased the nuclear abundance of MdCNR8 relative to that in *MdCNR8-FLAG*-transformed cells, whereas the overexpression of MdSIZ1 failed to promote the nuclear recruitment of MdCNR8^K39R^, which suggests that the K39 is required for SUMOylation of MdCNR8 and essential for enhancing its nuclear translocation (Figure 5A). In addition, we analyzed the abundance of MdCNR8 or MdCNR8^K39R^ in the absence of MdSIZ1. In the apple calli transformed with *antiMdSIZ1* combined with *MdCNR8-FLAG* or *MdCNR8^K39R^-FLAG*, MdCNR8 or MdCNR8^*K*39*R*^ is predominantly localized in the cell membrane compared with proteins fractioned from cytoplasm and nuclei (Figure 5B). These results indicated that SUMOylation of MdCNR8 is essential for its nuclear translocation and MdSIZ1 increases nuclear localization of MdCNR8.

To further confirm the biological function of MdCNR8 SUMOylation, MdCNR8-GFP or MdCNR8^*K*39*R*^-GFP was cotransformed with MdSIZ1-RFP into tobacco epidermal cells for subcellular localization analysis. The MdCNR8-GFP was mainly localized to cell membrane, and the expression of MdSIZ1-RFP greatly enhanced re-localization of MdCNR8-GFP into cell nuclei. The MdCNR8^K39R^-GFP was also mainly localized to cell membrane. However, cotransformation of MdSIZ1-RFP with MdCNR8^K39R^-GFP could not change its membrane-localization due to its SUMOylation site mutation (Figure 5C and Supplementary Figure 5). Collectively, these results suggested that SUMOylation could enhance nuclear localization of MdCNR8.

### MdSIZ1 Positively Regulates Cell Proliferation in the Root Meristematic Zone and Root Growth

**FIGURE 6:**
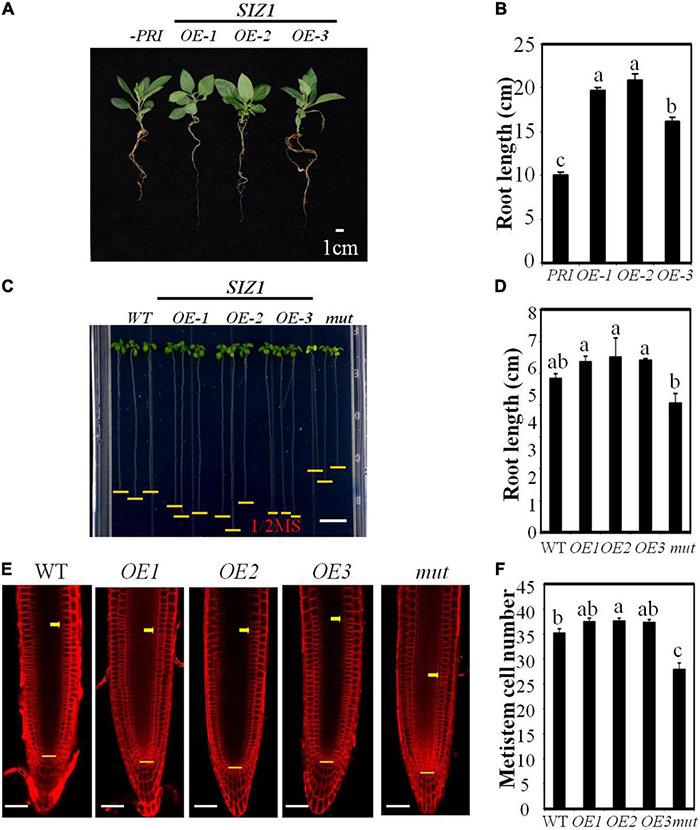
MdSIZ1 positively regulates cell proliferation in the root meristematic zone and root growth. **(A)** Phenotype of 40-day-old apple seedlings of *MdSIZ1* overexpression lines (OE-1, OE-2, OE-3) and the control line infected with empty vector. Bars = 1 cm. **(B)** Primary root length of 40-day-old apple seedlings in **(A)**. **(C)** Root phenotype of 7-day-old Arabidopsis seedlings of the WT, *MdSIZ1* overexpression (OE-1, OE-2, OE-3) and *atsiz1* mutant (*mut*). Bars = 1 cm. **(D)** Primary root length of the WT, OE-1, OE-2, OE-3, and mutant Arabidopsis seedlings in **(C)**. **(E)** Confocal microscopy images of the FM4-64-stained WT, *MdSIZ1* overexpression lines (OE-1, OE-2, OE-3) and *atsiz1* mutant (*mut*) Arabidopsis seedlings. Yellow arrows and horizontal lines indicate the approximate position of root meristem zone. Bars = 50 μm. **(F)** Meristem cell number of the WT, OE-1, OE-2, OE-3, and mutant Arabidopsis seedlings. The cell number of 7-day-old roots was counted and shown as averages ± SD (*n* = 5 to 10). Data in **B,D,F** are the means (*n* = 5 to 20) ± SD. Different letters on each bar denote significant differences (*p* < 0.01, ANOVA, Tukey’s correction).

To determine whether *MdSIZ1* functions in mediating organ size, we overexpressed *MdSIZ1-GFP* in apple root through *Agrobacterium rhizogenes* infection (Zhou et al., 2019). The strong fluorescent signal and gene expression analysis indicated that the transgene functioned well in apple roots (Supplementary Figure 6). We found that these plants overexpressing *MdSIZ1* exhibited longer roots than the plant infected with empty vector (Figures 6A,B), which indicated that *MdSIZ1* may promote root growth. Additionally, the overexpression of *MdSIZ1* in Arabidopsis also resulted in increased root elongation compared to the wide type (Zhang et al., 2016; Zhang et al., 2019; Zhou et al., 2019); by contrast, the Arabidopsis *siz1* mutant exhibited shorter roots than its WT (Figures 6C,D). To understand whether the enhanced root growth by *MdSIZ1* was derived from the alteration of cell proliferation, we examined the cell number in root meristematic zone. The results showed that there were more cells in root meristematic zone of *MdSIZ1* overexpression seedlings than the wide type, whereas the cell number in roots of the *siz1* mutant was much fewer (Figures 6E,F). These results indicated that MdSIZ1 positively mediated root growth by regulating cell proliferation in the root meristematic zone.

### MdSIZ1-Mediated SUMOylation of MdCNR8 Regulates Cell Proliferation and Root Growth in ARABIDOPSIS

Overexpression of MdCNR8 in Arabidopsis resulted in elongated roots promoted us to question whether MdCNR8 could be interacted by AtSCE1 for SUMOylation to regulate cell division. A Y2H assay showed that MdCNR8 was able to directly interact with AtSCE1 (Supplementary Figure 7), which suggests that MdCNR8 may be sumoylated in Arabidopsis.

**FIGURE 7:**
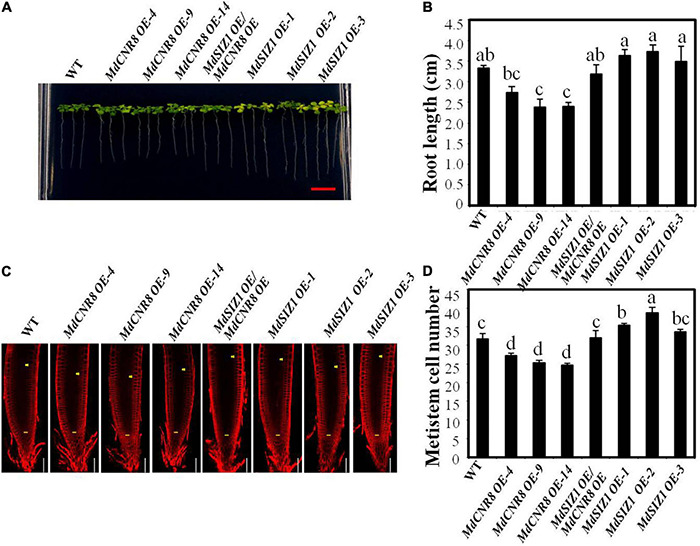
MdSIZ1-mediated SUMOylation of MdCNR8 regulates cell proliferation and root growth in Arabidopsis. **(A)** Root phenotype of 7-day-old Arabidopsis seedlings of the WT, *MdCNR8* overexpression lines (MdCNR8 OE-4, MdCNR8 OE-9, MdCNR8 OE-14), *MdSIZ1* overexpression lines (MdSIZ1 OE-1, MdSIZ1 OE-2, MdSIZ1 OE-3), and *MdCNR8-OE/MdSIZ1-OE* double overexpression line. Bars = 1 cm. **(B)** Primary root length of 7-day-old Arabidopsis seedlings of the WT, MdSIZ1 OE-1, MdSIZ1 OE-2, MdSIZ1 OE-3, MdCNR8 OE-4, MdCNR8 OE-9, MdCNR8 OE-14, and *MdCNR8-OE/MdSIZ1-OE* double overexpression line. Data are the means (*n* = 20) ± SD. Different letters on each bar denote significant differences (*p* < 0.05, ANOVA, Tukey’s correction). **(C)** Confocal microscopy images of the FM4-64-stained WT, *MdCNR8* overexpression lines (MdCNR8 OE-4, MdCNR8 OE-9, MdCNR8 OE-14), *MdSIZ1* overexpression lines (MdSIZ1 OE-1, MdSIZ1 OE-2, MdSIZ1 OE-3), and *MdCNR8-OE/MdSIZ1-OE* double overexpression Arabidopsis seedlings. Yellow arrows and horizontal lines indicate the approximate position of root meristem zone. Bars = 50 μm. **(D)** Meristem cell number of the WT, MdSIZ1 OE-1, MdSIZ1 OE-2, MdSIZ1 OE-3, MdCNR8 OE-4, MdCNR8 OE-9, MdCNR8 OE-14, and *MdCNR8-OE/MdSIZ1-OE* double overexpression Arabidopsis seedlings. Data are the means (*n* = 5) ± SD. Different letters on each bar denote significant differences (*p* < 0.01, ANOVA, Tukey’s correction).

To further confirm that SUMOylation of MdCNR8 in Arabidopsis will be required for regulating cell division, we expressed MdCNR8 in WT and MdSIZ1 overexpression Arabidopsis. Based on the *MdCNR8* and *MdSIZ1* transcript level determined by qRT-PCR, three *MdCNR8-GFP* Arabidopsis overexpression lines, three *MdSIZ1-GFP* Arabidopsis overexpression lines, and *MdCNR8-OE/MdSIZ1-OE* Arabidopsis double expression line were chosen for further analysis (Supplementary Figure 8). Overexpression of *MdCNR8* in WT Arabidopsis (*MdCNR8 OE-4*, *OE-9*, and *OE-14)* caused shorter roots than WT Arabidopsis, while the roots of MdSIZ1 overexpression lines (*MdSIZ1 OE-1*, *OE-2*, and *OE-3*) were longer than WT. However, the reduced root elongation by overexpression of *MdCNR8* could be rescued by overexpression of *MdSIZ1*, which results in that roots of the *MdCNR8-OE/MdSIZ1-OE* Arabidopsis double expression line were similar to the WT (Figures 7A,B). The microscopic examination revealed that the meristematic cell number of MdSIZ1 and MdCNR8 double overexpression line was significantly more than MdCNR8 overexpression lines but less than MdSIZ1 overexpression lines (Figures 7C,D). These results indicated that MdCNR8-mediated cell proliferation and root growth in Arabidopsis are dependent on its SUMOylation by MdSIZ1.

### MdSIZ1-Mediated SUMOylation of MdCNR8 Regulates Cell Proliferation and Organ Size in Apple

**FIGURE 8:**
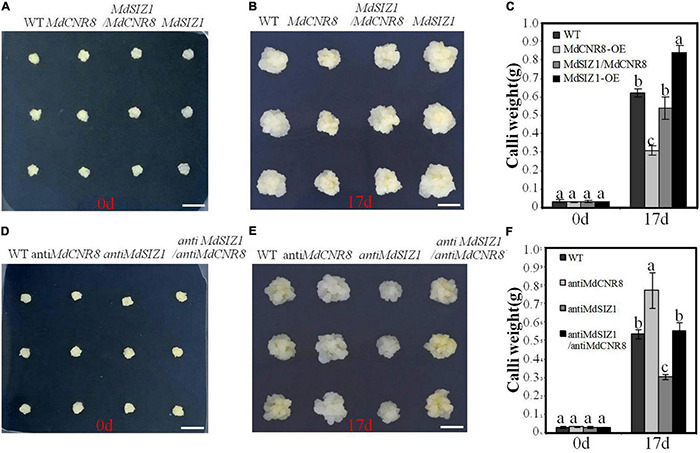
MdSIZ1-mediated SUMOylation of MdCNR8 regulates cell proliferation and organ size in apple. **(A–C)** Phenotypes of the WT, *MdCNR8* overexpression (*MdCNR8*), *MdSIZ1* overexpression (*MdSIZ1*), and *MdCNR8-OE/MdSIZ1-OE* double overexpression (*MdSIZ1/MdCNR8*) apple calli. Bars = 1 cm. The weight of 0-day-old calli and 17-day-old calli was measured and shown as averages ± SD (*n* = 11 to 20). **(D–F)** Phenotypes of the WT, *anti-MdCNR8, anti-MdSIZ1, anti-MdSIZ1/anti-MdCNR8* transgenic apple calli. Bars = 1 cm. The weight of 0-day-old calli and 17-day-old calli was measured and shown as averages ± SD (*n* = 11 to 20). Different letters on each bar denote significant differences (*p* < 0.01, ANOVA, Tukey’s correction).

To explore the role of MdSIZ1-mediated SUMOylation of MdCNR8 in apple,*MdCNR8*, *MdSIZ1*, and *MdSIZ1/MdCNR8* were expressed in apple calli. The overexpression of *MdCNR8* caused less cell proliferation and weight than WT calli, while the cell number and weight of MdSIZ1 overexpression calli were more than WT apple calli (Supplementary Figure 9 and Figures 8A–C). However, the reduced cell numbers and fresh weight by overexpression of *MdCNR8* could be rescued by the overexpression of *MdSIZ1*, which resulted in that cell number and fresh weight of the *MdCNR8-OE/MdSIZ1-OE* double expression apple calli were similar to the WT (Figures 8A–C). Moreover, silencing MdSIZ1 (anti-MdSIZ1) resulted in less cell proliferation and callus fresh weight compared to the WT control, whereas the calli of anti-MdCNR8 transgenic line exhibited higher fresh weight. As expected, the phenotype of anti-MdSIZ1 could be restored by simultaneously silencing MdCNR8 in anti-MdSIZ1/anti-MdCNR8 double silencing apple calli (Figures 8D–F). These results indicated that the regulation of cell proliferation by MdSIZ1 may be dependent on MdCNR8 in apple.

**FIGURE 9:**
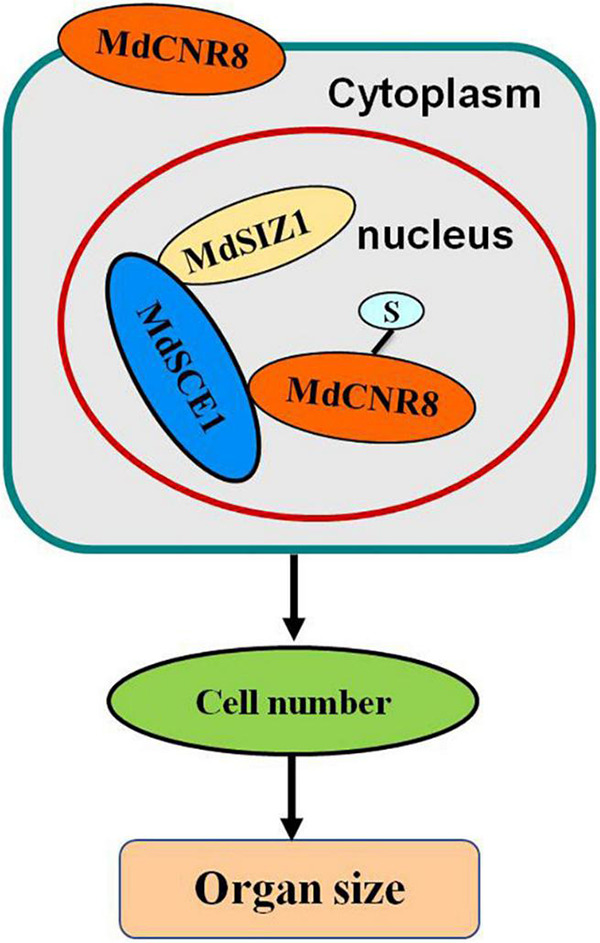
Model depicting how SUMOylation of MdCNR8 regulates MdSIZ1-mediated cell number and organ size in plants (S, SUMO).

Taken together, these results suggest that MdSIZ1-mediated SUMOylation of MdCNR8 regulates cell number, thereby affecting organ size in plants (Figure 9).

## Discussion

Organ size is one of the most important agronomic characteristics that have been long sought by plant breeders. It is generally believed that agricultural products with large organs (*e.g.*, large fruits) are associated with high yield and high market value. Cell number and cell size are the two key determinants for plant organ size. In this study, we have identified and isolated a *FW2.2*-like gene from apple, *MdCNR8*, and demonstrated that it could regulate fruit size and root growth through controlling cell proliferation (Figure 1). Regardless of the organ type such as fruit, nodule, root, and grain, the previous studies have suggested that FW2.2-like genes across different plant species played a conservative role in negatively controlling organ size (Frary et al., 2000; Dahan et al., 2010; Guo et al., 2010; Guo and Simmons, 2011; De Franceschi et al., 2013; Xu et al., 2013; Qiao et al., 2017). Although intensive efforts were given to understand the mechanism of FW2.2-like protein in regulating cell proliferation, knowledge on how FW2.2-like genes exert their role in controlling cell proliferation remains fragmented. FW2.2 has been extensively demonstrated to be a plasma membrane-anchored protein that may facilitate transporting ions (Song et al., 2004; Nakagawa et al., 2007), but there is no molecular evidence on how ion transport meditates cell division. A number of two independent studies have, respectively, placed an AGAMOUS-like gene (AG2) or a CKII in association with FW2.2 to control cell division, but it remains controversial on how FW2.2 interacts with either AG2 or CKII to exert its function since no evidence supported their direct interaction *in vivo*, despite that an *in vitro* interaction was detected (Li and He, 2015). In addition, in this study, we have cloned a homolog of CKII kinase (*MD04G1054100*, *MD07G1240200*, *MD02G1221400*, *MD06G1044800*, and *MD07G1094900*) from apple and performed Y2H assay to verify the interaction between MdCNR8 and MdCKII, but we have not observed a direct interaction between MdCNR8 and MdCKII. These results indicated that the CKII-mediated cell division pathway might not be applied to MdCNR8 in apple.

### SUMOylation Can Alter Cellular Localization of MdCNR8

In eukaryotes, protein function is determined by amino acids and also post-translational modifications such as SUMOylation and ubiquitination, which may impact function, activity, and cellular location of mature proteins. No study has been reported on whether any post-translational modification occurs to FW2.2-like proteins for exerting their roles in controlling organ size. In this study, we have observed that cellular localization of MdCNR8 can be altered by SUMOylation, which results in a translocation of this membrane-localized protein to the nucleus (Figures 5, 9).

SUMOylation and deSUMOylation are proposed to fine-tune the balance between nuclear, cytosolic, and membranous functions of targets through promoting shuttling of these targets into and out of the nucleus (Pichler and Melchior, 2002). The altered cellular localization due to SUMOylation has been widely reported in mammal cells. SUMOylation enabled mobilization of transmembrane fragment of a cell adhesion molecule L1 from plasma membrane to the nucleus for its function in the developing and adult nervous system (Lutz et al., 2012). Mutation of SUMOylation site at K1172 in L1 resulted in an abortion of this nuclear transport. Similarly, the membrane-associated insulin-like growth factor 1 receptor can be sumoylated at the three conserved lysine residues for its translocation to the nucleus to exert its roles in development and cancer biology (Sehat et al., 2010). In planta, the evidence from Arabidopsis and maize also suggested that SUMOylation may predominantly control nuclear event since the majority of canonical SUMOs and their conjugates are localized to the nucleus (Augustine and Vierstra, 2018). Accumulating evidence demonstrated the role of SUMOylation in altering cellular location in plants. Kim et al. (2018) showed that a nitrate reductase in Arabidopsis is mainly localized onto cytoplasmic membrane and that expression of AtSIZ1 affects subcellular localization of this protein, which results in its translocation to the nucleus (Kim et al., 2018). By contrast, the SUMOylation promotes cytoplasmic partitioning of NIb [RNA-dependent RNA polymerase of Turnip mosaic virus (TuMV)] and NPR1 (non-expressor of pathogenesis-related (PR) genes1), both of which are mainly localized in the nucleus prior to being sumoylated (Saleh et al., 2015; Cheng et al., 2017).

MdCNR8 lacks a nuclear localization sequence (NLS) and predominantly bound on the plasma membrane prior to SUMOylation as shown in Figure 5. Interaction with the NLS-containing SCE1/SIZ1 for its SUMOylation is essential for the nuclear translocation of MdCNR8 since the mutation of its SUMOylation site K39 caused the abortion of translocation that has been enhanced by expression of MdSIZ1 (Figure 5). It is still not clear how SUMOylation of MdCNR8 promotes this nuclear transport event and what roles the translocated MdCNR8 plays in the nucleus. It is speculative that the cytoplasmic-to-nuclear translocation may fulfill specific functions of MdCNR8 in the cell nucleus, for example, formation of a protein complex as a transcriptional modulator. This assumption may be consolidated by the results reported by Li et al. (2017a). An endoplasmic reticulum localized B-CELL LYMPHOMA 2-ASSOCIATED ATHANOGENE 7 (AtBAG7) protein in Arabidopsis is translocated into the nucleus only after being sumoylated under heat stress (Li et al., 2017b), and this translocated AtBAG7 could directly interact with a transcription factor WRKY29. Protein interaction with WRKY29 and heat tolerance were abolished when two SUMOylation residues K179 and K212 of AtBAG7 were both mutated, which suggests that SUMOylation and translocation are required for the AtBAG7-WRKY29 interaction and regulation of downstream target genes (Li et al., 2017b). Similarly, the SUMOylation of NPR1 is also required for its interacting with a TGA transcription activator for PR gene expression to tightly control plant immune responses, but the SUMOylation-deficient NPR1 mutant can only interact with another WRKY transcription repressor.

### SIZ1 Controls Cell Growth and Organ Size

Genes required for post-translational modification have been widely reported to contribute to controlling of plant organ size by promoting cell proliferation or expansion. The ubiquitin-binding protein DA1 and the E3 ubiquitin-ligase BIG BROTHER (BB) in Arabidopsis are the two plant growth repressors that can control sizes of seed and other organs by limiting cell proliferation in early stage of organogenesis (Li et al., 2008). A recent report also showed that the Arabidopsis SUMO E3 Ligase AtMMS21 controls root growth by mediating cell proliferation *via* the E2Fa/DPa pathway (Huang et al., 2009; Liu et al., 2016). SUMOylation enhances the dissociation of the E2Fa/DPa transcription factor complex for cell cycle regulation. Similarly, the loss of function Arabidopsis mutant *siz1* has a dwarf phenotype with decreased number and size of cells compared to the WT (Figure 6) (Catala et al., 2007; Miura et al., 2010). In addition, Miura et al. (2005) reported that SIZ1 is involved in the regulation of root growth in response to phosphate starvation (Miura et al., 2005). However, different mechanisms were previously proposed for SIZ1 to control plant growth. Catala et al. (2007) proposed that the dwarf phenotype of *siz1-3* Arabidopsis mutant may be attributed to the reduced expression of genes involved in brassinosteroid biosynthesis and signaling since knockout of these genes caused a similar dwarf phenotype to *siz1-3* (Catala et al., 2007). On the other hand, the increment in salicylic acid has been suggested to be linked with the dwarf phenotype of *siz1-3* Arabidopsis mutant (Miura et al., 2010), while the inhibition of primary root growth in *siz1-3* mutant in response to phosphate starvation was derived from alteration of auxin accumulation (Miura et al., 2011). All regulatory modules proposed in the previous studies involve different plant hormones which may participate in regulating various biological processes. In this study, we have demonstrated that MdSIZ1 may regulate organ size through a specific MdCNR8-dependent cell division pathway. Despite extensive studies on its conserved role in regulating organ size, our study demonstrated concrete evidence in how post-translational modification by SUMO process affects plant growth; these results may also bridge the knowledge gap in understanding the molecular mechanism by which how SUMOylation affects plant development in response to various stresses.

## Data Availability Statement

The original contributions presented in the study are included in the article/Supplementary Material, further inquiries can be directed to the corresponding author.

## Author Contributions

C-XY, Y-JH, and G-LW conceived and designed the experiments. G-LW, X-SS, C-LZ, and Y-LZ performed the research. G-LW and Y-JH analyzed the data. G-LW, C-XY, and H-QH wrote the manuscript. All authors read and approved the manuscript. All authors contributed to the article and approved the submitted version.

## Conflict of Interest

The authors declare that the research was conducted in the absence of any commercial or financial relationships that could be construed as a potential conflict of interest.

## Publisher’s Note

All claims expressed in this article are solely those of the authors and do not necessarily represent those of their affiliated organizations, or those of the publisher, the editors and the reviewers. Any product that may be evaluated in this article, or claim that may be made by its manufacturer, is not guaranteed or endorsed by the publisher.

## Footnotes

1
http://signal.salk.edu/tdnaprimers.2.html

2
http://www.abgent.com/sumoplot
