# Supplementary material for: The SUMO E3 Ligase MdSIZ1 Sumoylates a Cell Number Regulator MdCNR8 to Control Organ Size
Source: Front Plant Sci. 2022 Apr 15;13:836935. doi: 10.3389/fpls.2022.836935 (PMC9051543; doi:10.3389/fpls.2022.836935)
Supplement: Supplementary file 1 [file Data_sheet_1.docx]

Supplementary Material

# Supplementary Figures and Tables

## Supplementary Figures


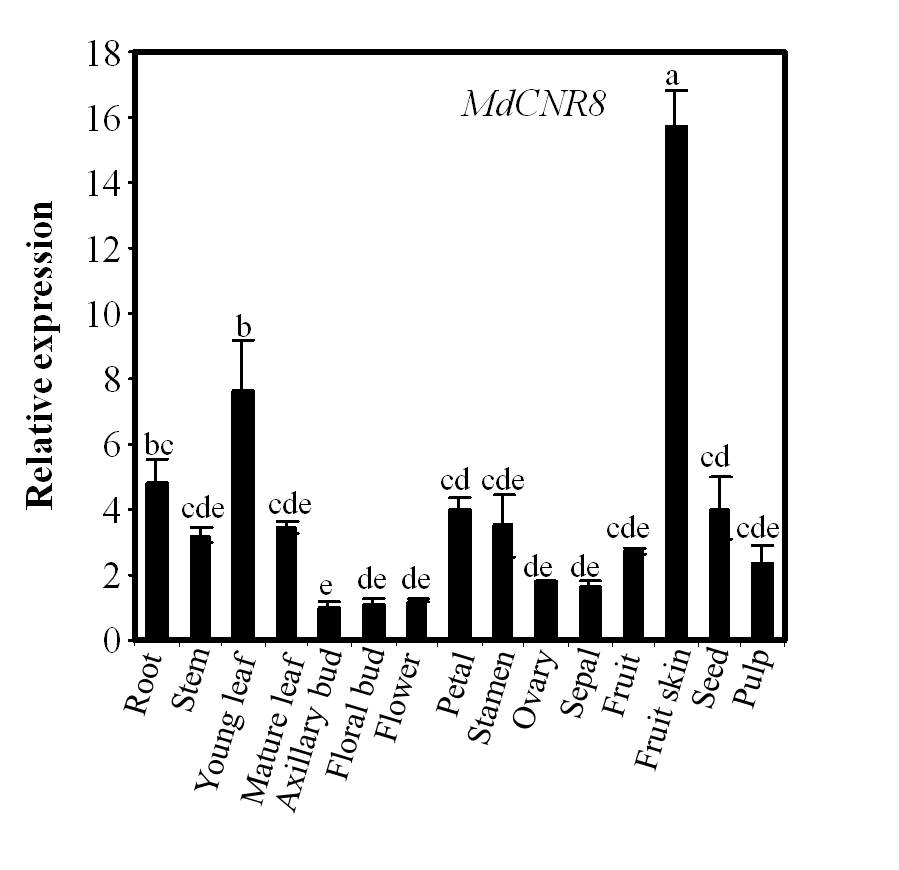


**Supplementary Figure 1.** **Expression profile of MdCNR8****in various apple tissues.**

Transcript level of *MdCNR8* in various apple tissues by qRT-PCR. Data are the means ± SD of three biological repeats. *MdACTIN* was used as an internal control. Different letters on each bar denote significant differences. (P<0.01, ANOVA, Tukey correction).


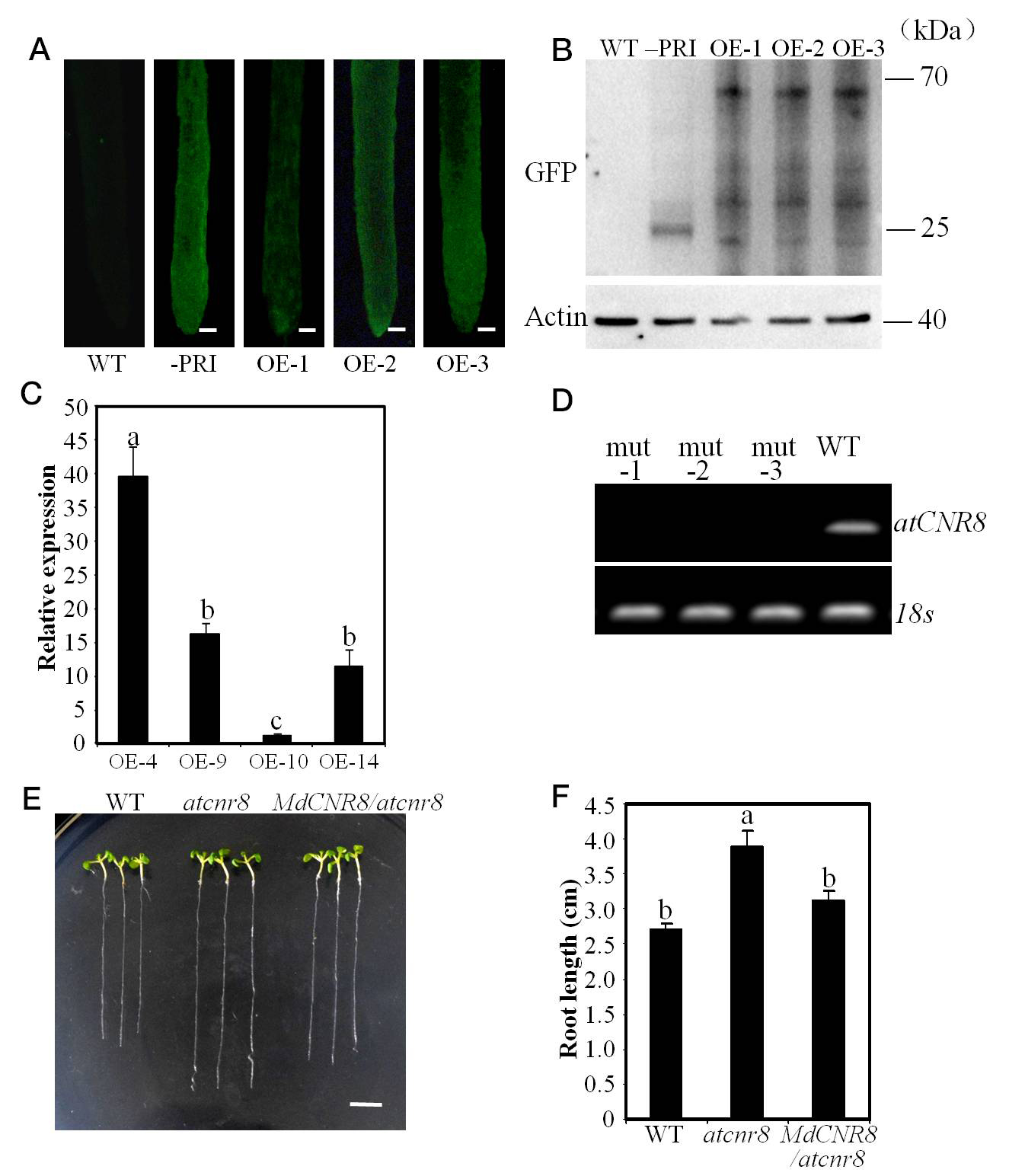


**Supplementary Figure 2.** **Expression level of *CNR8* and phenotype of *atcnr8*.**

**(A)** Fluorescent detection in roots of *MdCNR8* overexpression lines. Note: the stronger fluorescent signal of GFP observed in the transgenic apple roots compared to the wild type root. Bars = 50 μm. (B) Protein level of MdCNR8 in the wide type, the control line infected with empty vector and MdCNR8-overexpression lines (OE-1, OE-2, OE-3). The roots of 40-d-old seedlings were harvested for protein extraction and Western-blotting detection was performed with GFP and Actin antibody. ACTIN acts as the internal control. **(C)** Quantitative RT-PCR of *MdCNR8* in the *Arabidopsis thaliana*. Data are the means ± SD of three biological repeats. *At18s* was used as an internal control. Different letters on each bar denote significant differences (P<0.01, ANOVA, Tukey correction). **(D)** Transcript level of *AtCNR8* in the *Arabidopsis thaliana* T-DNA insertion mutant (*SALK_042402*) by RT-PCR. *18s gene* was used as an internal control. **(E)** Root phenotypes of the wild type (WT), *mutant* (*atcnr8*) and hybrid line of *MdCNR8*-overexpression and *atcnr8* mutant (*MdCNR8*/*atcnr8*) grown for 7 d on 1/2 MS medium. Bars = 5mm. **(F)** Primary root length of the WT, *atcnr8* and *MdCNR8*/*atcnr8*. The length of 7-d-old roots was measured and shown as averages ± SD (n = 11 to 20). Different letters on each bar denote significant differences (P<0.01, ANOVA, Tukey correction).


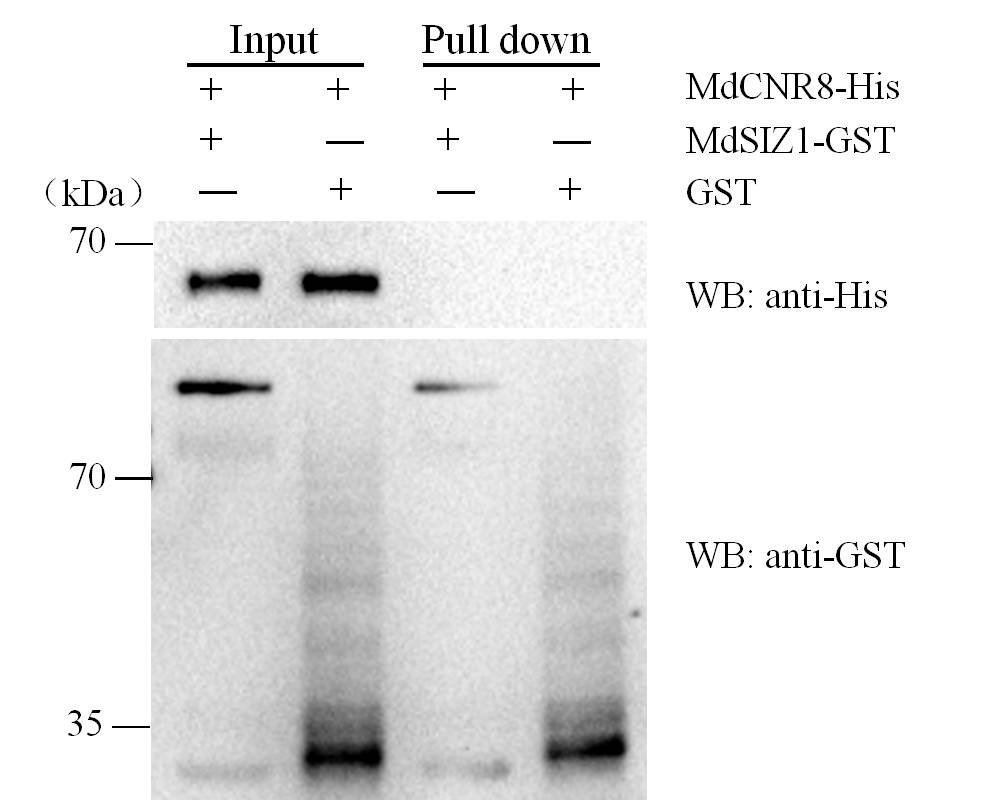


**Supplementary Figure 3.** **Physical interactions between MdSIZ1 and MdCNR8.**

Pull-down assays. Escherichia coli-expressed GST or MdSIZ1-GST proteins were incubated with a cobalt chelate affinity resin containing the immobilized histidine-tagged MdCNR8 protein. The proteins were detected via immunoblotting with anti-His and anti-GST antibody.


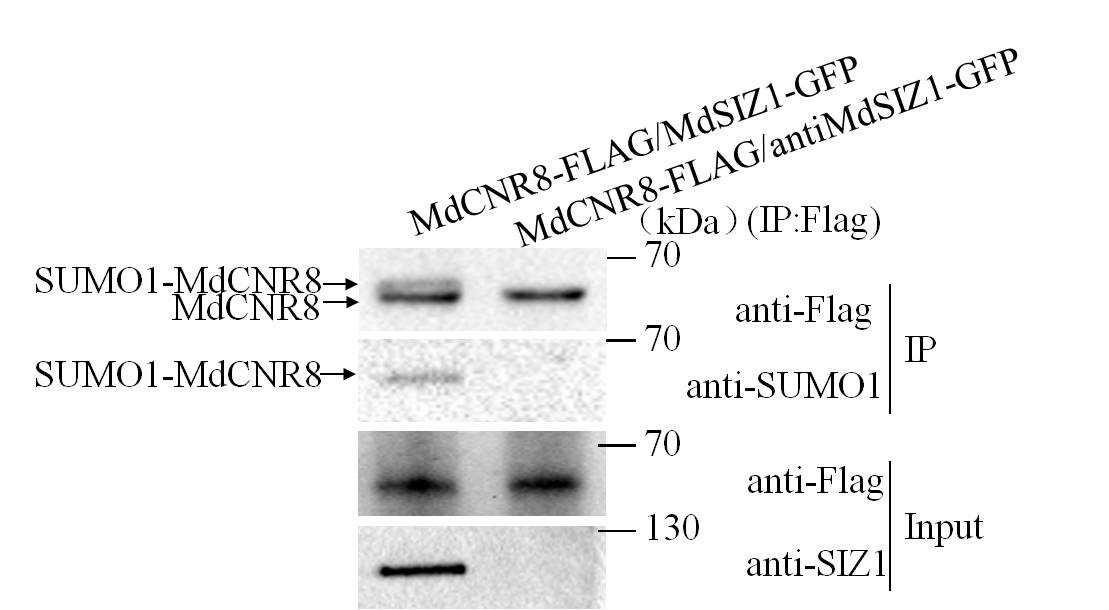


**Supplementary Figure 4.** **Sumoylation of MdCNR8 with or without MdSIZ1.**

MdSIZ1 SUMOylated MdCNR8 protein in vivo. Input: the whole-cell lysates before treatment. IP, soluble pellet fraction.


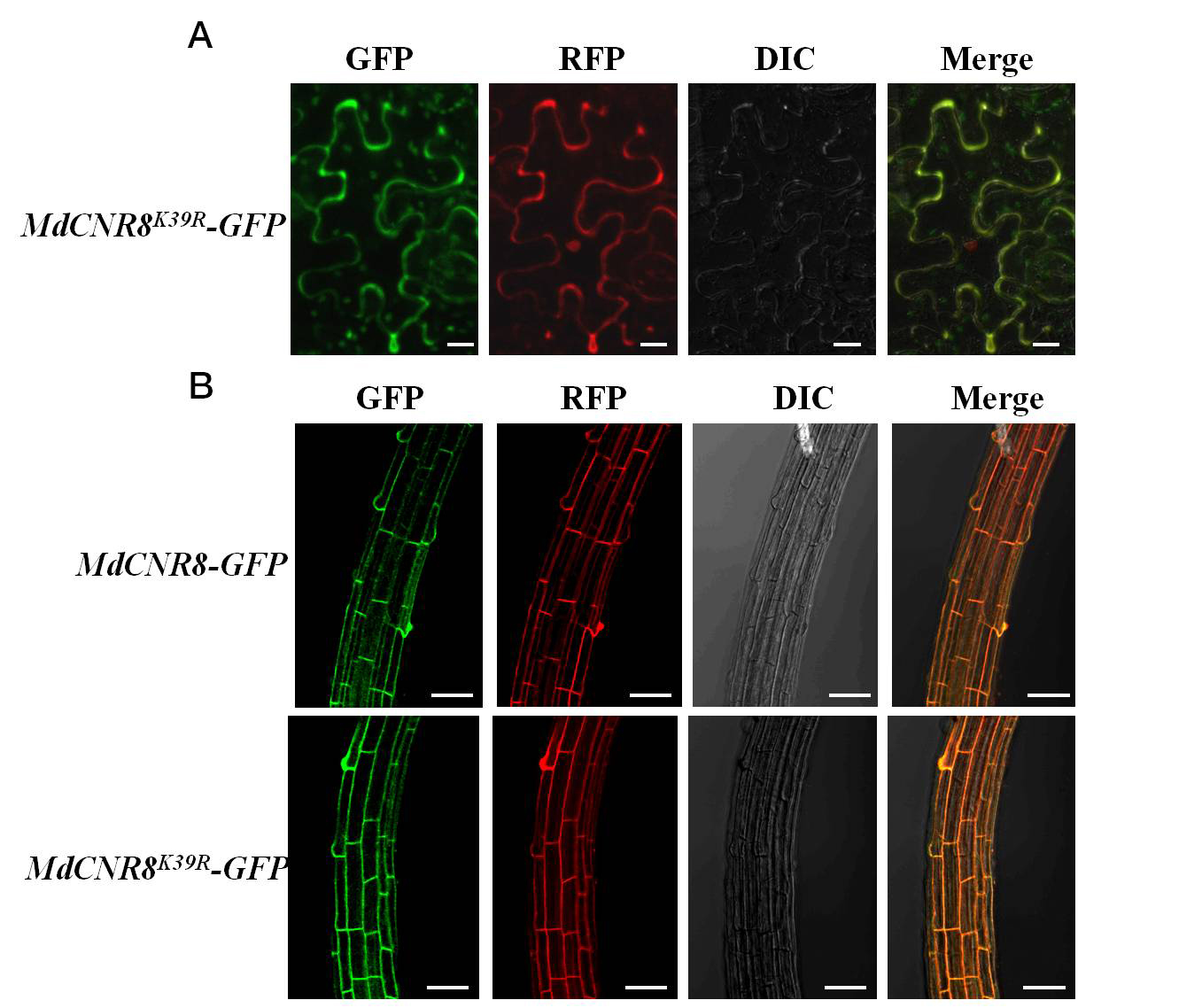


**Supplementary Figure 5.** Fluorescent detection of MdCNR8 or MdCNR8^K39R^-GFP subcellular localization.

**(A)** Fluorescent detection of MdCNR8^K39R^-GFP subcellular localization in tobacco leaves. Bars = 20 μm. **(B)** Fluorescent detection of MdCNR8 or MdCNR8^K39R^-GFP subcellular localization in Arabidopsis. Bars = 50 μm. GFP, Green ﬂuorescent protein; RFP, Red ﬂuorescent protein; DIC, bright field.


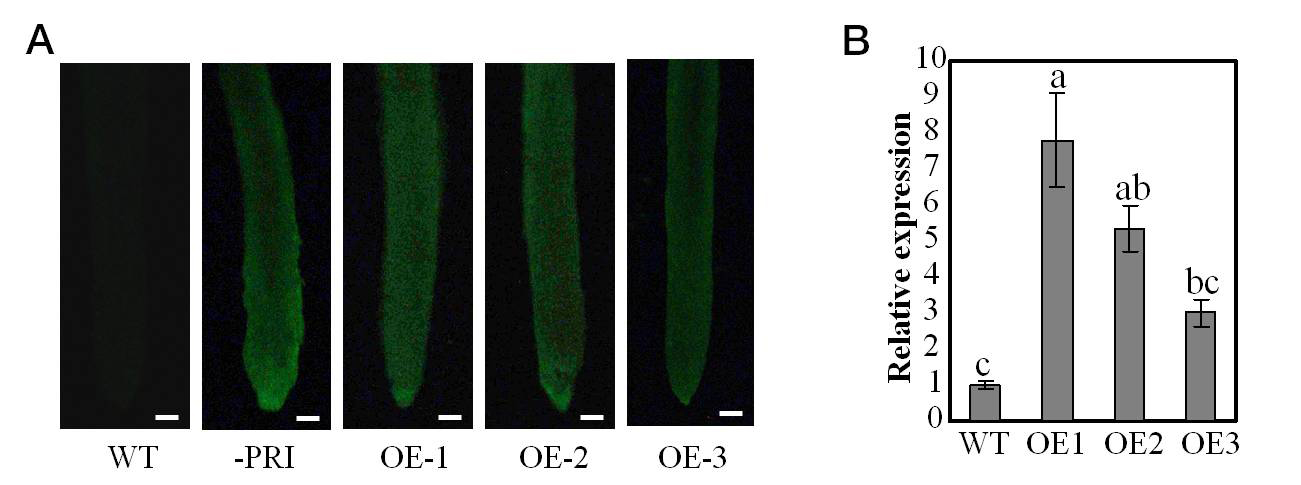


**Supplementary Figure 6.** **Expression level of *MdSIZ1*** **in apple roots overexpressing *MdSIZ1* infected with *Agrobacterium rhizomes.***

**(A)** Fluorescent detection in roots of *MdSIZ1* overexpression lines. Note: the stronger fluorescent signal of GFP observed in the transgenic apple roots compared to the wild type root. Bars = 50 μm. **(B)** Quantitative RT-PCR of *MdSIZ1* in the apple roots overexpressing *MdSIZ1*. Data are the means ± SD of three biological repeats. *MdACTIN* was used as an internal control. Different letters on each bar denote significant differences (P<0.01, ANOVA, Tukey correction).


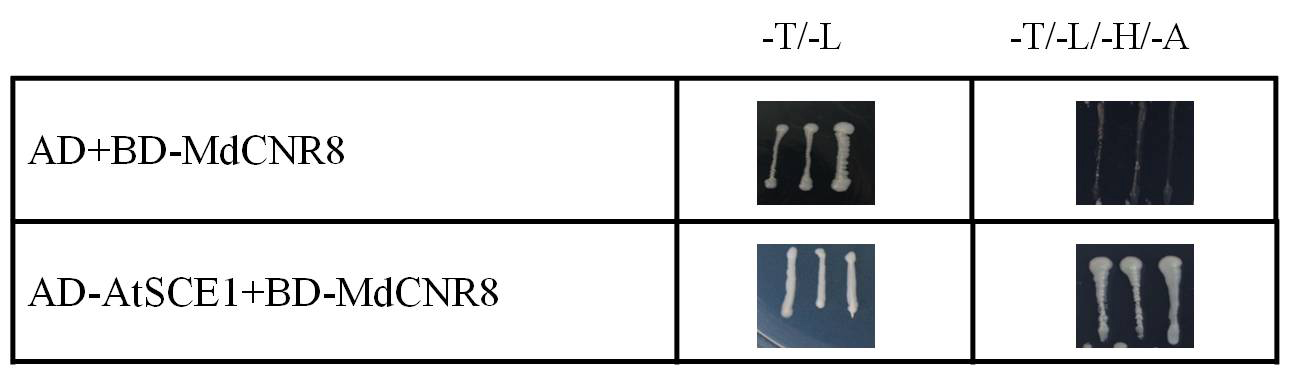


**Supplementary Figure 7.** **AtSCE1 interacts with MdCNR8 not AtCNR8.**

Y2H assays showed the interaction of AtSCE1 and MdCNR8. The combination of AD and BD-MdCNR8 was used as a negative control to indicate that MdCNR8 could not be activated autonomously. SD/ -T/ -L was for lacing Trp and -Leu in minimal SD base as transformation control. SD/ -T/ -L/ -H/ -A was for lacing Trp, Leu, His, and Ade in minimal SD base as a screen.


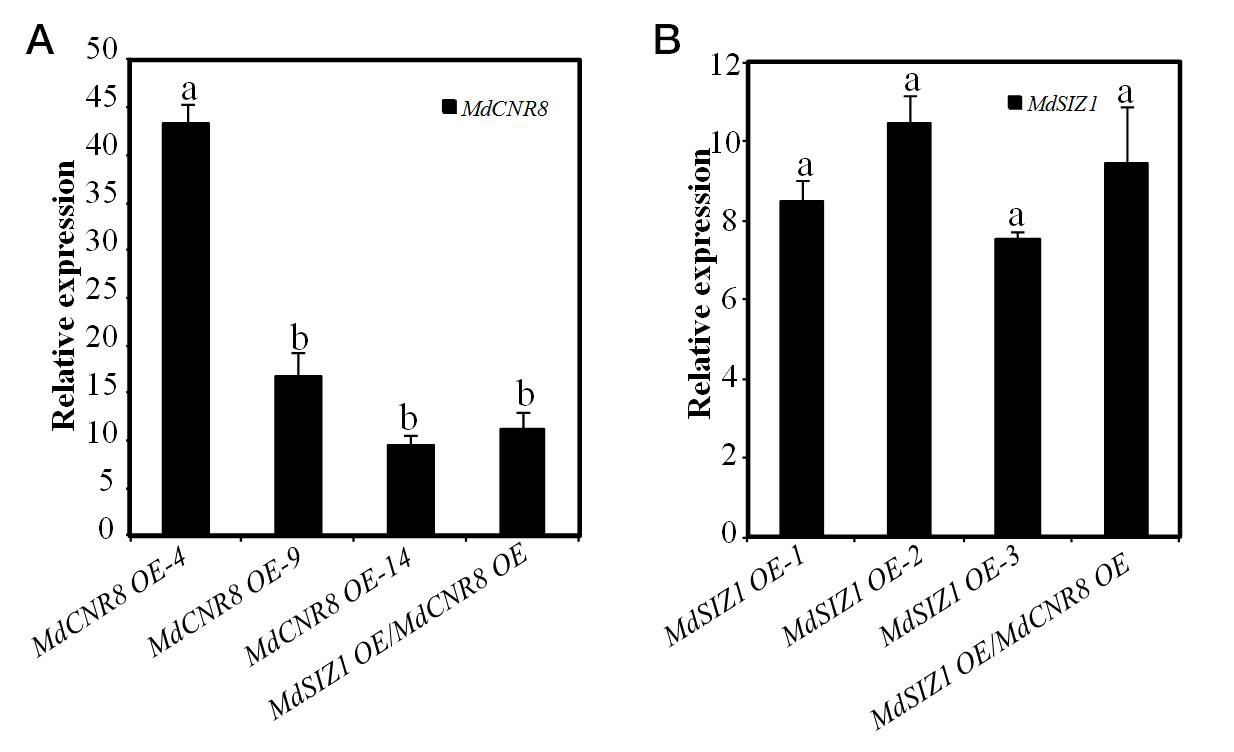


**Supplementary Figure 8.** **Expression level of *MdCNR8* and *MdSIZ1* in transgenic *Arabidopsis thaliana***

**(A-B)** Quantitative RT-PCR of *MdCNR8* and *MdSIZ1* in *Arabidopsis thaliana*. Data are the means ± SD of three biological repeats. *At18s* was used as an internal control. Different letters on each bar denote significant differences (P<0.01, ANOVA, Tukey correction).


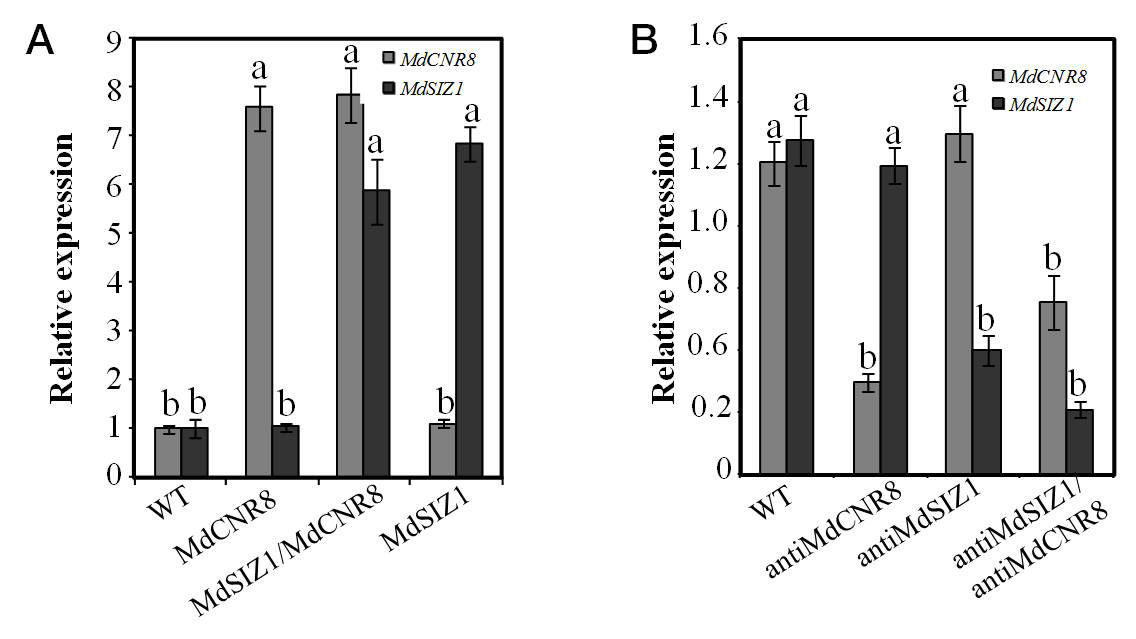


**Supplementary Figure 9.** **Expression level of *MdCNR8* and *MdSIZ1* in transgenic apple calli**

Quantitative RT-PCR of *MdCNR8* and *MdSIZ1* in transgenic calli. Data are the means ± SD of three biological repeats. *MdACTIN* was used as an internal control. Different letters on each bar denote significant differences (P<0.01, ANOVA, Tukey correction).

## Supplementary Tables

| Gene Prime name Prime sequence | | |
| --- | --- | --- |
| *CNR-PRI* | CNR-PRI-F | GTCGACATGGCGACCAACAACAGGGAGAGCA |
|  | CNR-PRI-R | GGTACCAGCTCCACGGCCCATGGCCTGCTCC |
| *MdCNR8-PET32a* | MdCNR8-PET32a-F | TCGAGCTCCGTCGACAAATGGCGACCAACAACAGGGAGAGCA |
|  | MdCNR8-PET32a-R | GTGGTGGTGCTCGAGAAGCTCCACGGCCCATGGCCTGCTCC |
| *Y3H-BD-SIZ1-N* | Y3H-BD-SIZ1-N-F | ACTGTATCGCCGGAATTCCCGGGAATGGATTTGGTAGCTAGTTGCAAG |
|  | Y3H-BD-SIZ1-N-R | CCGGAATTAGCTTGGCTGCAGACATCTTTGAAGTGATACGGTTG |
| *Y3H-BD-CNR8* | Y3H-BD-CNR8-F | ACTGTATCGCCGGAATTCCCGGGAATGGCGACCAACAACAGGGA |
|  | Y3H-BD-CNR8-R | CCGGAATTAGCTTGGCTGCAGAAGCTCCACGGCCCATGGCCTGC |
| *Y3H-brige-SCE1* | Y3H-brige-SCE1-F | AAGAAGAGAAAGGTGGCGGCCGCAATGTCTGGAGGTATAGCTCGCGG |
|  | Y3H-brige-SCE1-R | CATGGGAGATCAGCCCGAAGATCTCACAAGAGGTGGGTATTGCCTGGA |
| *QRT-CNR* | QRT-CNR-F | AGGTGGTAATGGGTGCGTGT |
|  | QRT-CNR-R | TGCTACAGAACTCATCATTACGCCCAAGAC |
|  | 402--LP | CTCCAAGGAAGAAGAAGCCTC |
| *SALK_042402* | 402--RP | AGAGAGACTTGGGTCTGCTCC |
|  | LBb1.3 | ATTTTGCCGATTTCGGAAC |
| *RT-AtCNR8* | RT-AtCNR8-F | ATGGCGGGAGAAGATAGCCGT |
|  | RT-AtCNR8-R | CTTACGACCCATGGTTTGTTC |
| *CNR-FLAG* | CNR-FLAG-F | TCTAGAATGGCGACCAACAACAGGGAGAGCA |
|  | CNR-FLAG-R | GGTACCAGCTCCACGGCCCATGGCCTGCTCC |
| *CNR-BIFC* | CNR-BIFC-F | TCTAGAATGGCGACCAACAACAGGGAGAGCA |
|  | CNR-BIFC-R | GTCGACAGCTCCACGGCCCATGGCCTGCTCC |
| *JmCNR* | JmCNR-F | GAATTCATGGCGACCAACAACAGGGAGA |
|  | JmCNR-R | GTCGACAAGCTCCACGGCCCATGGCCT |
| *TOPO* | TOPO-F | GGTGAAGGGCTCCTTCTTAAAG |
|  | TOPO-R | AAGGGTGGGCGCGC |
| *CNR8-C-AD* | CNR8-C-AD-F | GAATTCTGTAGCAGCGATCTCGAAGTTTGTC |
|  | CNR8-C -AD-R | GTCGACAAGCTCCACGGCCCATG |
| *CNR8-N-AD* | CNR8-N-AD-F | GAATTCATGGCGACCAACAACAGGGA |
|  | CNR8-N -AD-R | GTCGACAGAACTCATCATTACGCCCAAGACAAGCAA |
| *AtSCE1-AD* | AtSCE1-AD-F | GAATTCATGGCTAGTGGAATCGCTC |
|  | AtSCE1-AD-R | GTCGACAGACAAGAGCAGGATACTGC |
| *MD04G1054100* | MD04G1054100-F | CAAAAAAAGAGATCGATGTACAGAGAGCGAGGGGTGGTT |
|  | MD04G1054100-R | GGATCCCCGGGAATTTGGCTTGTGAAGCTTGAATCCAAACAC |
| *MD07G1240200* | MD07G1240200-F | CAAAAAAAGAGATCGATGCCTAAAGATCAGAGAGGTGTAA |
|  | MD07G1240200-R | GGATCCCCGGGAATTTTCGTTGCGGACTTTGAAGCCGTGT |
| *MD02G1221400* | MD02G1221400-F | CAAAAAAAGAGATCGATGTATAGGGAACGTGGCGGTGGAGGCGTT |
|  | MD02G1221400-R | GGATCCCCGGGAATTCGGCTTGTGGAGCTTGAAACCAAAAACTCT |
| *MD06G1044800* | MD06G1044800-F | CAAAAAAAGAGATCGATGCACAGAGAGCGAGGGTTGGGT |
|  | MD06G1044800-R | GGATCCCCGGGAATTTGGCTTGTGAAGCTTGAATCCGAA |
| *MD07G1094900* | MD07G1094900-F | CAAAAAAAGAGATCGATGTATAGGGAACGTGGCGGTGGAGGCGGTGGCGGGT |
|  | MD07G1094900-R | GGATCCCCGGGAATTCGGCTTGTGGAGCTTGAAACCAAAAAC |
| *MdSCE1-BD* | MdSCE1-BD-F | CGGGATCCAAATGTCTGGAGGTATAG |
|  | MdSCE1-BD-R | GCGTCGACACACAAGAGGTGGGTA |
| *MdSCE1-GST* | MdSCE1-GST-F | CGGGATCCATGTCTGGAGGTATAG |
|  | MdSCE1-GST-R | GCGTCGACACACAAGAGGTGGGTA |
| *N-YFP-MdSCE1* | N-YFP-MdSCE1-F | TCTAGAATGTCTGGAGGTATAGCTCGC |
|  | N-YFP-MdSCE1-R | GTCGACCACAAGAGGTGGGTATTGCCT |
| *MdSCE1-RFP* | MdSCE1-RFP-F | AAGGAGCCCTTCACCATGTCTGGAGGTATAGCTCG |
|  | MdSCE1-RFP-R | GGCGCGCCCACCCTTCACAAGAGGTGGGTATTGCC |
| *N-YFP-MdSIZ1* | N-YFP-MdSIZ1-F | ACTAGTATGGATTTGGTAGCTAGTTGCA |
|  | N-YFP-MdSIZ1-R | GTCGACTTCAGAGTCCGAGTCAATAG |
| *QRT-MdSIZ1* | QRT-MdSIZ1-F | CAGTCCTTTCTCATTTCCTCG |
|  | QRT-MdSIZ1-R | TCTAACGCTTCATCTCGTCAG |
| *SlACTIN* | *Sl*ACTIN-F | TGTGACAATGGAACTGGAATGG |
|  | *Sl*ACTIN-R | CCATACCCACCATCACACCAG |
| *At18s* | At18s-F | GCTCTGGCTTGCTCTGATG |
|  | At18s-R | CTGCCTTCCTTGGATGTGGTAG |
| *MdACTIN* | MdACTIN-F | AGGCGCGAAATTACCAATCC |
|  | MdACTIN-R | GCCCTCCAATTGTTCCTCGTTAAG |
